# Prevalence and characteristics of people with a high body mass index across the kidney disease spectrum: a population-based cohort study

**DOI:** 10.23889/ijpds.v9i5.2734

**Published:** 2024-09-18

**Authors:** Gurleen Sahi, Jennifer Reid, Michael Chiu, Louise Moist, Kyla Naylor, Saverio Stranges, Kamel Omer, Amanda Vinson, Janet Madill, Kristin K Clemens

**Affiliations:** 1 Schulich School of Medicine and Dentistry, Western University; 2 ICES; 3 ICES Western; 4 Division of Nephrology, Department of Medicine, London Health Sciences Centre, Western University; 5 Division of Nephrology, Department of Medicine, Schulich School of Medicine and Dentistry, Western University; 6 Department of Epidemiology and Biostatistics, Western University; 7 Lawson Health Research Institute and London Health Sciences Centre; 8 Faculty of Medicine at Dalhouse; 9 Brescia University College, Western University; 10 Schulich School of Medicine and Dentistry, Western University

The prevalence of obesity is growing globally and has major health implications, particularly for those with chronic kidney disease (CKD). People with obesity have a higher risk of CKD progression and face barriers to transplantation. Studies on the epidemiology and outcomes of obesity in the Canadian CKD population are lacking.

This population-based cohort study included adults aged 18 and over with a hospital encounter in London, Ontario, Canada from 2010 to 2019 where height and weight were recorded. They were stratified into CKD stages 1-5 (by estimated glomerular filtration rate (eGFR)), dialysis or renal transplant status and by body mass index (BMI) using Canada Obesity Guidelines. Outcomes included prevalence of BMIs by CKD stage, and CKD progression and transplant outcomes across BMI classes.

Our cohort included 198,151 patients. The prevalence of BMI ≥30 kg/m2 grew as eGFR declined (i.e., 37% in stage 1, 41.5% in stage 3b, 40.9% in stage 4) but fell in end-stages (i.e., 37.4% in Stage 5, 38.8% in dialysis, 38.5% in transplant recipients). CKD progression and kidney failure appeared more frequent in those with a BMI ≥30 kg/m2. In a smaller sample, we noted that end-stage patients with a BMI ≥30 kg/m2 were less frequently transplanted, but experienced post-transplant complications less often.

This study revealed that high BMI is a prevalent issue in the Canadian CKD population and may influence kidney outcomes and transplant candidacy. This data will help inform clinical trials to create and study weight loss interventions for those with CKD and obesity.

